# ProSIMSIt: The Best of Both Worlds in Data-Driven Rescoring and Identification Transfer

**DOI:** 10.1021/acs.jproteome.4c00967

**Published:** 2025-03-22

**Authors:** Firas Hamood, Wassim Gabriel, Pia Pfeiffer, Bernhard Kuster, Mathias Wilhelm, Matthew The

**Affiliations:** † Chair of Proteomics and Bioanalytics, School of Life Sciences, Technical University of Munich, 85354 Freising, Germany; ‡ Assistant Professorship of Computational Mass Spectrometry, School of Life Sciences, Technical University of Munich, 85354 Freising, Germany; § Munich Data Science Institute (MDSI), Technical University of Munich, 85748 Garching, Germany

**Keywords:** isobaric labeling, missing values, spectrum clustering, identification transfer, data-driven rescoring, peptide-spectrum match, phosphoproteomics, drug-response profiling

## Abstract

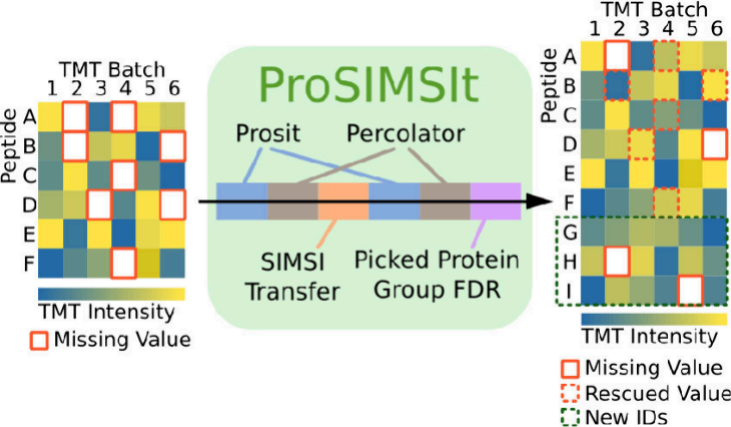

Multibatch isobaric labeling experiments are frequently applied for clinical and pharmaceutical studies of large sample cohorts. To tackle the critical issue of missing values in such studies, we introduce the ProSIMSIt pipeline. It combines the advantages of tandem mass spectrum clustering via SIMSI-Transfer and data-driven rescoring via Prosit and Oktoberfest. We demonstrate that these two tools are complementary and mutually beneficial. On large-scale cancer cohort data, ProSIMSIt increased the number of peptide spectrum matches (PSMs) by 40% on both global and phosphoproteome data sets. Furthermore, on data from proteome-wide drug-response profiling of post-translational modifications (decryptM), our pipeline substantially increased drug–PTM relations and revealed previously unseen downstream effects of drug target inhibition. ProSIMSIt is available as an open-source Python package with a simple command line interface that allows easy application to MaxQuant result files.

## Introduction

Isobaric labeling techniques such as tandem mass tags (TMTs) are frequently employed in proteomics to multiplex samples, allowing for the simultaneous measurement of up to 35 different conditions.^1^ These techniques are particularly valuable in large-scale cancer cohorts,^2^ substantially reducing the time required to process hundreds or even thousands of samples. Additionally, they are frequently used to measure the proteomic response of a system along dose, time, or temperature dimensions.^3−5^ A key advantage of isobaric labeling is that missing values are rare within the individual batches. However, when combining data from multiple batches, variations in the peptides identified and quantified across batches lead to a growing issue of missing values.^6^

To address this missing value problem, we previously introduced SIMSI-Transfer.^7^ This tool utilizes the fragment ion spectrum clustering algorithm MaRaCluster^8^ to find highly similar MS2 spectra across multiple experiments and leverages this information to transfer peptide identifications. A limitation of this approach is that it does not directly control the false discovery rate (FDR), as the transferred identifications lack search engine scores. This limitation can be addressed by leveraging a seminal advancement in peptide identification, i.e., data-driven rescoring with tools such as MS2rescore^9^ and Oktoberfest.^10^ These tools compute similarity scores between experimentally observed and in silico-predicted peptide properties,^11−14^ such as fragment ion intensities and retention time, and use these scores as additional features in Percolator^15^ to rerank the peptide identifications and estimate the FDR. In fact, this approach has proven so potent that—aside from large search spaces like immunopeptidomics and meta-proteomics—gains are now mainly limited by search engines failing to provide a correct peptide candidate for an MS2 spectrum. This can, for example, be due to errors in the monoisotopic precursor mass assignment or low spectrum quality. Data-driven rescoring of the transferred peptide spectrum matches (PSMs) from SIMSI-Transfer elegantly addresses the limitations of the two individual approaches. It provides additional high-quality peptide candidates to the rescoring approach and directly estimates the FDR on them.

Here, we present ProSIMSIt, a pipeline for multibatch isobaric labeling experiments that integrates SIMSI-Transfer with Prosit and Percolator to (1) reduce the number of missing values, (2) increase the number of identified peptides via rescoring, and (3) control the FDR. We validated this approach using data from an endometrial carcinoma cohort study by the Clinical Proteomic Tumor Analysis Consortium (CPTAC),^16^ resulting in an increase of 41% peptide-spectrum matches (PSMs) at 1% FDR for the global proteome data set and 42% for the phosphoproteome data set compared to the original MaxQuant results. Additionally, we applied ProSIMSIt to a decryptM dose-dependent phosphoproteome experiment, which investigated the effects of six kinase inhibitors on the phosphoproteome of A549.^3^ Our pipeline generated 17% additional regulated phosphosite curves, substantially enhancing the comparability between the drugs and revealing previously overlooked downstream effects of the kinase inhibitions. ProSIMSIt is available via GitHub (https://github.com/kusterlab/ProSIMSIt); it is provided as a simple command line interface, allowing easy application to MaxQuant results.

## Experimental Section

### Data Sets

We utilized two data sets from a study supplied by CPTAC^17^ to benchmark ProSIMSIt, which includes one global proteome data set and one phosphoproteome data set. The study consisted of endometrial carcinoma samples collected from 95 patients measured in 17 TMT batches. TMT10plex labeling was used for isobaric labeling, where the initial nine channels contained patient samples, and the final channel held a common reference sample (“bridge channel”). Samples from individual patients were often measured multiple times across various batches or within different TMT channels of the same batch. The global proteome samples underwent deep fractionation into 24 fractions, yielding a total of 16.4 million MS2 spectra, while the phosphoproteome samples were fractionated into 12 fractions, resulting in 8.6 million MS2 spectra. The raw data can be accessed through Proteomic Data Commons identifiers PDC000125 (global proteome) and PDC000126 (phosphoproteome).

For assessing the benefit of ProSIMSIt for biological interpretations, we utilized a data set from decryptM.^3^ The decryptM approach applies an increasing dose of drugs across TMT channels to generate dose-resolved response curves of post-translational modification (PTM) peptides. We selected a six-kinase inhibitor data set, where the lung adenocarcinoma cell line A549 was treated with AZD8055 (mTOR inhibitor), dactolisib (PI3K and mTOR inhibitor), pictilisib (PI3Kα/δ inhibitor), dasatinib (BCR/ABL and Src inhibitor), nintedanib (VEGFR, FGFR, PDGFR inhibitor), and tideglusib (GSK3 inhibitor). The drugs were applied in doses between 10 μM and 1 nM and treated for 1 h. All mass spectrometry files of this data set are accessible from the ProteomeXchange Consortium via the PRIDE repository with the data set identifier PXD037285.

### Database Searching via MaxQuant

Each data set underwent comprehensive analysis utilizing MaxQuant version 1.6.17.0. The searches were conducted employing the default parameters of TMT 10plex (CPTAC) or 11plex (decryptM); trypsin was used as the enzyme for in silico digestion with a maximum of two missed cleavages, additionally permitting cleavages prior to proline residues. Cysteine carbamidomethylation was used as a fixed modification, whereas methionine oxidation and N-terminal acetylation were added as variable modifications. Match between Runs was not used for any data set, as prior research showed inconsistent results in conjunction with isobaric labeling data for this MaxQuant version.^7^ For the analysis of the phosphoproteome data, serine, threonine, and tyrosine phosphorylation were added as variable modifications. Mass tolerances of 20 ppm for precursor ions during the initial search, 4.5 ppm for the primary search, and 20 ppm for the MS/MS fragment ions were implemented. The CPTAC data sets were searched against a reference proteome sourced from UniProt in August 2020. An updated reference proteome was retrieved in August 2023 and utilized for the decryptM data set. For further processing, a PSM-level output file containing the highest-scoring peptide for each spectrum was used. The resulting data was filtered to achieve a 1% false discovery rate (FDR) on both peptide and protein levels for the direct assessment of MaxQuant results and for further processing using SIMSI-Transfer. Additionally, for each data set, another search with the same parameters was conducted retaining all results without applying an FDR filter, which was used as the input for Oktoberfest and ProSIMSIt. msms.txt, msmsScans.txt, and allPeptides.txt files were used for downstream processing using Oktoberfest, SIMSI-Transfer, and ProSIMSIt.

### Oktoberfest

All Oktoberfest runs were performed using version 0.6.2, available via GitHub (https://github.com/wilhelm-lab/oktoberfest). To generate retention time and fragment intensity predictions, a publicly accessible instance of Koina was used (https://koina.proteomicsdb.org/). For the global proteome rescoring, the previously published models *Prosit_2020_intensity_TMT* and *Prosit_2020_irt_TMT* were used for fragment intensity and retention time prediction, respectively. For the phosphoproteome data, two novel soon-to-be published Prosit models were used, namely, *Prosit_2024_intensity_PTMs_gl* and *Prosit_2024_irt_PTMs_gl*. The models are already available for public usage via Koina, and we provide an overview of the model performance in Figure S1.

### SIMSI-Transfer

The latest release of SIMSI-Transfer, version 0.6.1, was obtained from GitHub (https://github.com/kusterlab/SIMSI-Transfer) and used for the isolated SIMSI-Transfer runs. Each run was performed with the recommended settings “--stringencies 10 --maximum_pep 5” for the clustering stringency and maximum posterior error probability for transfers, respectively. SIMSI-Transfer uses MaRaCluster to generate clusters of MS2 spectra with high similarity. In brief, MaRaCluster utilizes a rarity-based distance metric, which calculates spectral similarity by weighting “rare” fragment peaks higher than more common ones. This follows the idea that peaks shared by few spectra offer more evidence than peaks shared by a large number of spectra. The generated pairwise distance matrix is then used to perform complete-linkage hierarchical clustering, grouping MS2 spectra of high similarity. SIMSI-Transfer then combines the identification information gained from MaxQuant with the cluster information from MaRaCluster and uses the clusters to transfer peptide identifications from identified to unidentified spectra in each cluster. The exact parameters of SIMSI-Transfer were optimized to generate PSMs with approximately 1% FDR in the original SIMSI-Transfer publication.

The MaxQuant msms.txt, msmsScans.txt, and allPeptides.txt files at 100% FDR were filtered down to 1% FDR as input for SIMSI-Transfer. The generated p10_msms.txt files were used to compare the results with those of MaxQuant, Oktoberfest, and ProSIMSIt.

Combining the cluster information from MaRaCluster with the identifications gained by MaxQuant can generate clusters with spectra identified as conflicting peptides. We therefore also added a functionality that utilizes ambiguous clusters in ProSIMSIt. Previously, whenever an ambiguous or PTM-isomeric cluster, i.e., clusters with two or more conflicting peptide identifications, was encountered, no identifications were transferred. Instead, with the new “--ambiguity_decision keep_all” argument, each unidentified spectrum in this cluster is assigned multiple PSMs corresponding to the conflicting peptide identifications in the cluster. The result can be considered equivalent to the “second peptide” option that several database search tools provide.

### The ProSIMSIt Pipeline

**Figure 1:**
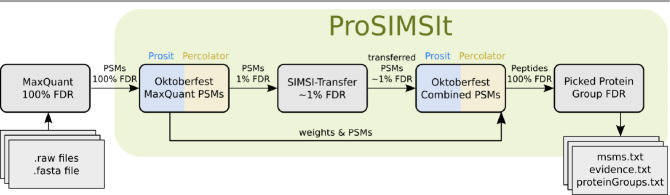
Data flow in the ProSIMSIt pipeline. Gray: input, processing step, or output; green: ProSIMSIt pipeline; blue: Prosit; yellow: Percolator.

An overview of the pipeline is presented in Figure 1 and Figure S2. Additionally, an in-depth explanation of every processing step, including input- and output files as well as FDR filtering steps, is provided in Text S1 and S2. As input, the ProSIMSIt pipeline requires a MaxQuant run with 100% peptide-level FDR and 100% protein-level FDR. MaxQuant’s msms.txt and the raw files are used as input for Oktoberfest (“Oktoberfest MaxQuant PSMs”). This includes: (1) conversion from proprietary .raw format to .mzML using the ThermoRawFileParser (https://github.com/compomics/ThermoRawFileParser), (2) peptide property prediction utilizing Prosit via Koina,^18^ and (3) FDR estimation by Percolator.^15^ The outputs are PSM-level output files for both targets and decoys, and the support vector machine weights obtained by Percolator.

As input for SIMSI-Transfer, the PSMs are filtered at 1% FDR and converted into a MaxQuant msms.txt format. SIMSI-Transfer is then executed with the recommended settings “--stringencies 10 --maximum_pep 5” as well as “--ambiguity_decision keep_all” (see the SIMSI-Transfer section above)

Identifications gained by SIMSI-Transfer did not undergo peptide property prediction at this stage. Therefore, after filtering for those identifications and making the SIMSI-Transfer output compatible with Oktoberfest, the transferred PSMs are submitted to Prosit for peptide property prediction (“Oktoberfest Combined PSMs” in Figure 1). To select the best PSM per spectrum, the percolator input files of the two Oktoberfest runs are combined. In this combined file, PSMs gained by SIMSI-Transfer replace PSMs for the same spectrum if the latter PSM did not make the 1% FDR of the first Oktoberfest run.

In the final step of the ProSIMSIt pipeline, Percolator estimated FDRs for all PSMs using the predicted peptide properties as features. To reduce runtime, the Percolator feature weights of the initial Oktoberfest run are reused in this step as a static model. Note that Percolator retains the best scoring PSM for each spectrum, e.g., those for which the “--ambiguity_decision keep_all” resulted in multiple PSMs. Additionally, we investigated the presence of systematic effects caused by SIMSI-Transfer that could change the score distribution of targets and decoys (Suppl. Figure S3). To assess if the minor shift in the score distribution affects the FDR estimation, we additionally performed an entrapment experiment (Text S2, Figure S4). The PSM-level output files are then converted into MaxQuant’s evidence.txt format and submitted to Picked Protein Group FDR^19^ v0.7.2 for protein inference and protein-group-level FDR filtering. This results in ProSIMSIt output files on the PSM, peptide, and protein levels for further downstream processing.

Processing the 408 raw file global proteome CPTAC data set with the ProSIMSIt pipeline took a total of 7:50 h utilizing 12 threads on an AMD EPYC 7452 Processor, excluding conversion into .mzML format.

### Curve Classification

We utilized CurveCurator^20^ to classify the TMT reporter intensities of each PSM into upgoing, downgoing, or nonregulated curves. To prevent the default behavior of CurveCurator aggregating curves on the peptide level, we added an additional “Name” column into the input msms.txt file, resulting in PSM-level curves. The resulting classifications were used to evaluate the differences between the observed curves from MaxQuant and ProSIMSIt.

### Analysis of decryptM Data

For analyzing the decryptM results on phosphosite level, we annotated all PSMs with the open source tool pSite annotation v0.5.3 (https://github.com/kusterlab/psite_annotation) using a .fasta database retrieved from PhosphoSitePlus on January 2024 as the input. This tool adds various phosphosite-specific information to each modified peptide sequence, including the exact position of the site in the canonical sequence of each protein (e.g., AKT2 S474 representing a phosphorylation of serine 474 of the AKT2 protein). The six PI3K/AKT/mTOR-related phosphosites^21−26^ KS6B1 S427, AKT2 S474, LARP1 S774, AKTS1 S202, RS6 S244, and RS6 S235 were selected for further downstream analysis.

### Comparison of Results

The prediction of intensity and retention time by Prosit is limited to peptides with specific characteristics. Therefore, all comparisons between tools were performed after filtering for peptides in accordance with the limitations of the *Prosit_2020_intensity_TMT* model: (1) length of 7–30 amino acids, (2) maximum charge state of 6+, (3) not containing the proteinogenic but nongenomically encoded amino acids selenocysteine or pyrrolysine, and (4) no N-terminal protein acetylation.

All comparisons are performed between PSMs filtered at a 1% FDR wherever applicable. In the case of SIMSI-Transfer, all generated PSMs are considered, as the parameters for SIMSI-Transfer were optimized to maintain an FDR of approximately 1%.

## Results and Discussion

### Overview of the ProSIMSIt Pipeline

ProSIMSIt combines the advantages of SIMSI-Transfer, Prosit, and Percolator into one pipeline. As input, a MaxQuant analysis performed at 100% FDR is required. First, Prosit and Percolator are run inside Oktoberfest to provide the benefits of Prosit rescoring. Next, SIMSI-Transfer transfers peptide identifications between batches. This includes a new feature in SIMSI-Transfer that transfers multiple peptide candidates for spectra in clusters with conflicting peptide identifications. These transferred identifications are then evaluated in a second Oktoberfest step alongside the PSMs generated in the first Oktoberfest run. Finally, the rescored PSMs are submitted to the Picked Protein Group FDR package for protein group identification and quantification.

### Oktoberfest and SIMSI-Transfer Yield Complementary Identifications

**Figure 2:**
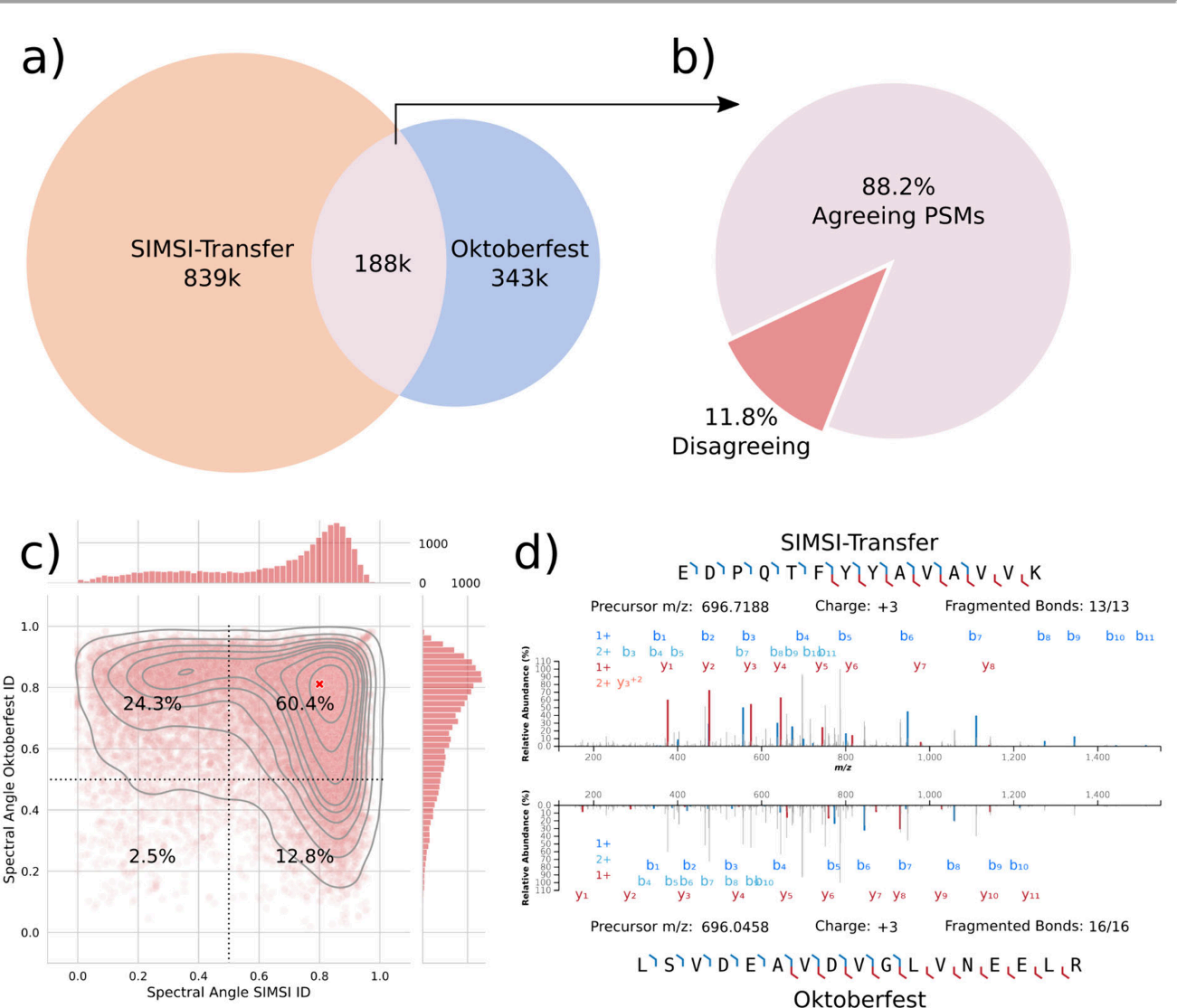
Comparison of PSMs generated by SIMSI-Transfer and Oktoberfest. (a) Venn diagram of spectra identified by Oktoberfest and SIMSI-Transfer. (b) Agreement of PSMs identified by both Oktoberfest and SIMSI-Transfer individually. (c) Spectral angle between predicted and measured spectrum for SIMSI-Transfer and Oktoberfest PSMs of the same spectrum, with the spectrum from (d) highlighted in red. (d) Example mirror plot of one spectrum with two conflicting identifications from SIMSI-Transfer (top) and Oktoberfest (bottom).

The rationale for combining Oktoberfest and SIMSI-Transfer stemmed from the observation that the additional identifications produced by each of the tools exhibited limited overlap. To demonstrate this complementarity, we applied both tools to the CPTAC endometrial carcinoma global proteome data set,^16^ which comprises 17 TMT batches with 24 raw files each. Of the 531 000 and 1 027 000 additional spectra identified by Oktoberfest and SIMSI-Transfer respectively, only 188 000 were identified by both tools (Figure 2a). Closer investigation revealed that only 17% of the PSMs generated by SIMSI-Transfer had the same peptide identification in the MaxQuant 100% FDR output. As this output is used for rescoring by Oktoberfest, which can verify or discard only hypothetical PSMs from such an unfiltered run, the remaining 83% PSMs were impossible to identify using Oktoberfest.

Within the 188 000 PSMs in the overlap, 88.2% were identified as the same peptide by both tools, while 11.8% had differing peptide identifications (Figure 2b). We further examined these 22 000 spectra with conflicting peptide identifications between the two tools. To analyze these discrepancies, we calculated spectral angle values between the measured MS2 spectrum and the predicted spectrum by Prosit for each of the identifications. A spectral angle of 1 indicates that the two spectra are identical, while an angle of 0 signifies no overlap. Notably, more than 60% of these spectra showed a spectral angle greater than 0.5 for both peptide identifications (Figure 2c). This suggests that these spectra are chimeric, i.e., the conflicting peptide identifications arise from coisolated peptides in the mass spectrometer, with each tool identifying one of these, as exemplified in Figure 2d and Figure S5.

### ProSIMSIt Achieves Large Gains in Cohort Studies

**Figure 3:**
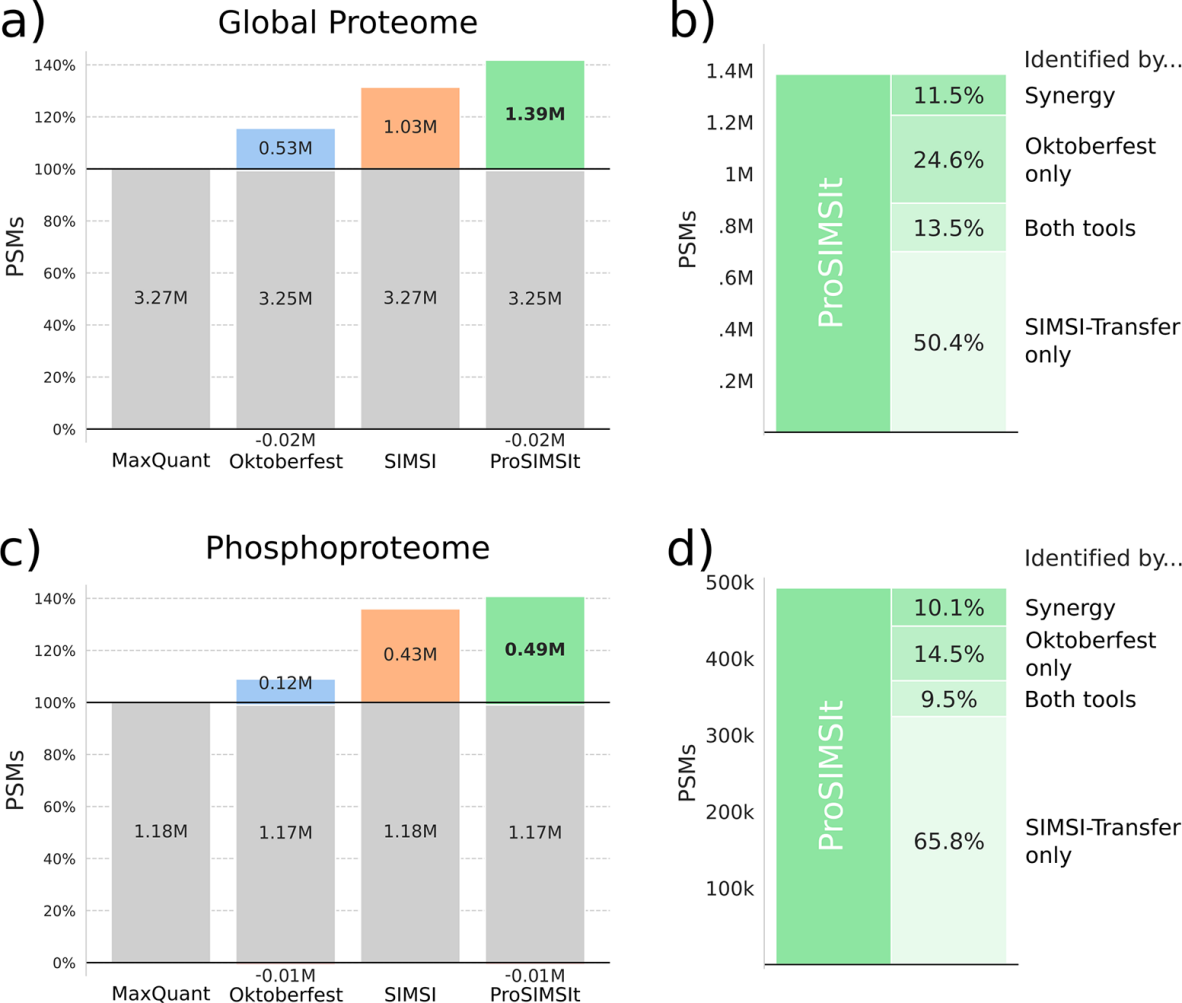
Identified PSMs gained by SIMSI-Transfer, Oktoberfest, and ProSIMSIt. (a) PSMs gained and lost by applying SIMSI-Transfer, Oktoberfest, and ProSIMSIt on the CPTAC endometrial carcinoma global proteome data set. (b) Zoom-in into PSMs gained by ProSIMSIt and distribution of identification sources. (c) Same as (a), but for the phosphoproteome data set. (d) Same as (b), but for the phosphoproteome data set.

We applied ProSIMSIt along with Oktoberfest and SIMSI-Transfer individually to reanalyze the MaxQuant results of the CPTAC endometrial carcinoma global proteome and phosphoproteome data sets. For the global proteome, MaxQuant identified 3 300 000 PSMs, SIMSI-Transfer yielded 1 030 000 additional PSMs (+31%), and Oktoberfest provided 560 000 additional PSMs (+16%) but also removed 20 000 PSMs. ProSIMSIt outperformed both individual tools, identifying 1 390 000 (+42%) additional PSMs while also only removing 20 000 (Figure 3a). On the peptide-level, ProSIMSIt increased the overall identifications by 11% compared to MaxQuant, from 236 000 to 262 000. Due to the SIMSI-Transfer step only allowing for the transfer of already identified peptides to other spectra, this gain is not as high as on the PSM level. Of the PSMs gained by ProSIMSIt, 50.4% were identified by SIMSI-Transfer and 24.6% were identified by Oktoberfest when used independently (Figure 3b). Additionally, 13.5% were identified by both tools, whereas the remaining 11.5% resulted from the synergy between the two, primarily consisting of transferred identifications from novel identifications obtained by Oktoberfest. On the phosphoproteome, MaxQuant identified 1 180 000 PSMs, whereas ProSIMSIt identified 490 000 (+42%) additional PSMs, outperforming the 430 000 (+36%) additional PSMs identified by SIMSI-Transfer alone (Figure 3c). The additional PSMs of ProSIMSIt are mainly driven by SIMSI-Transfer; still, 10.1% of the PSMs are generated from the synergy between the two tools (Figure 3d). On the peptide level, ProSIMSIt increased the number of identifications by 14%, from 144 000 to 164 000.

Importantly, ProSIMSIt has the added benefit over SIMSI-Transfer that it estimates the PSM-level FDRs. SIMSI-Transfer used the proportion of clusters containing conflicting peptide identifications as a proxy for the global FDR. This left doubt for individual transferred PSMs, for example, if the original cluster only contained a single PSM with a high posterior error probability. The PSM-level FDR of ProSIMSIt is particularly advantageous for phosphoproteomic analyses, where individual phosphosites are often of primary interest. More generally, the overall performance and observed synergy of ProSIMSIt show that SIMSI-Transfer and Oktoberfest are complementary and mutually beneficial.

Current research has investigated the accuracy of established FDR filtering approaches, which could potentially affect ProSIMSIt in multiple ways. Conservatively controlling the FDR on a peptide level is easier than on spectrum level due to the occurrence of multiple PSMs for the same peptide.^27^ Specifically for Percolator, multiple PSMs for a single peptide can additionally cause problems because the same peptide can potentially be used for both training and testing. This would lead to data leakage, which could lead to overfitting and an underestimation of the FDR during the following target-decoy competition.^28^ To assess how strongly ProSIMSIt is affected by those issues, we performed an entrapment experiment, calculating entrapment FDRs for MaxQuant, Oktoberfest, and ProSIMSIt (Text S3). At a set FDR filter of 1%, both Oktoberfest and ProSIMSIt showed a lower limit of 0.85% and an upper limit of 1.06% eFDR at 1% FDR, indicating a similarly accurate FDR control compared to 0.84% lower limit and 1.05% upper limit from MaxQuant (Suppl. Figure S4). In a future version of ProSIMSIt, it might make sense to employ further tools like Percolator-RESET^28^ to ensure a rigid FDR control with data sets of any kind.

### ProSIMSIt Enhances Interpretability of Drug Perturbation Experiments

An experimental setup particularly well-suited for ProSIMSIt involves a single cell line or tissue with multiple batches that each explore a different perturbation or condition while varying a variable such as dose, time, or temperature within each batch. In this setting, the transfer of identifications across batches becomes especially effective, as each peptide is likely present in each batch, and beneficial, as reduced missing values for the same peptide enable better comparisons. For example, in decryptM,^3^ each additional PSM reveals the behavior of a post-translational modification (PTM) site upon treatment, making it highly valuable for deciphering the molecular mode of action of the drugs. This also highlights the advantage of the proper FDR estimation procedure of ProSIMSIt.

To illustrate these benefits, we applied ProSIMSIt to a data set from decryptM, which involved treating the A549 cell line with six kinase inhibitors. Among the drugs studied, AZD8055, dactolisib, and pictilisib inhibit the PI3K/mTOR pathway, whereas dasatinib (BCR/ABL and Src), nintedanib (VEGFR), and tideglusib (GSK3) have other primary targets. To facilitate the biological interpretation of the dose–response curves, we applied CurveCurator,^20^ which uses a statistical model to classify curves as upregulated, downregulated, or not regulated. This way, we could confidently identify phosphopeptides regulated upon kinase inhibitor treatment.

**Figure 4:**
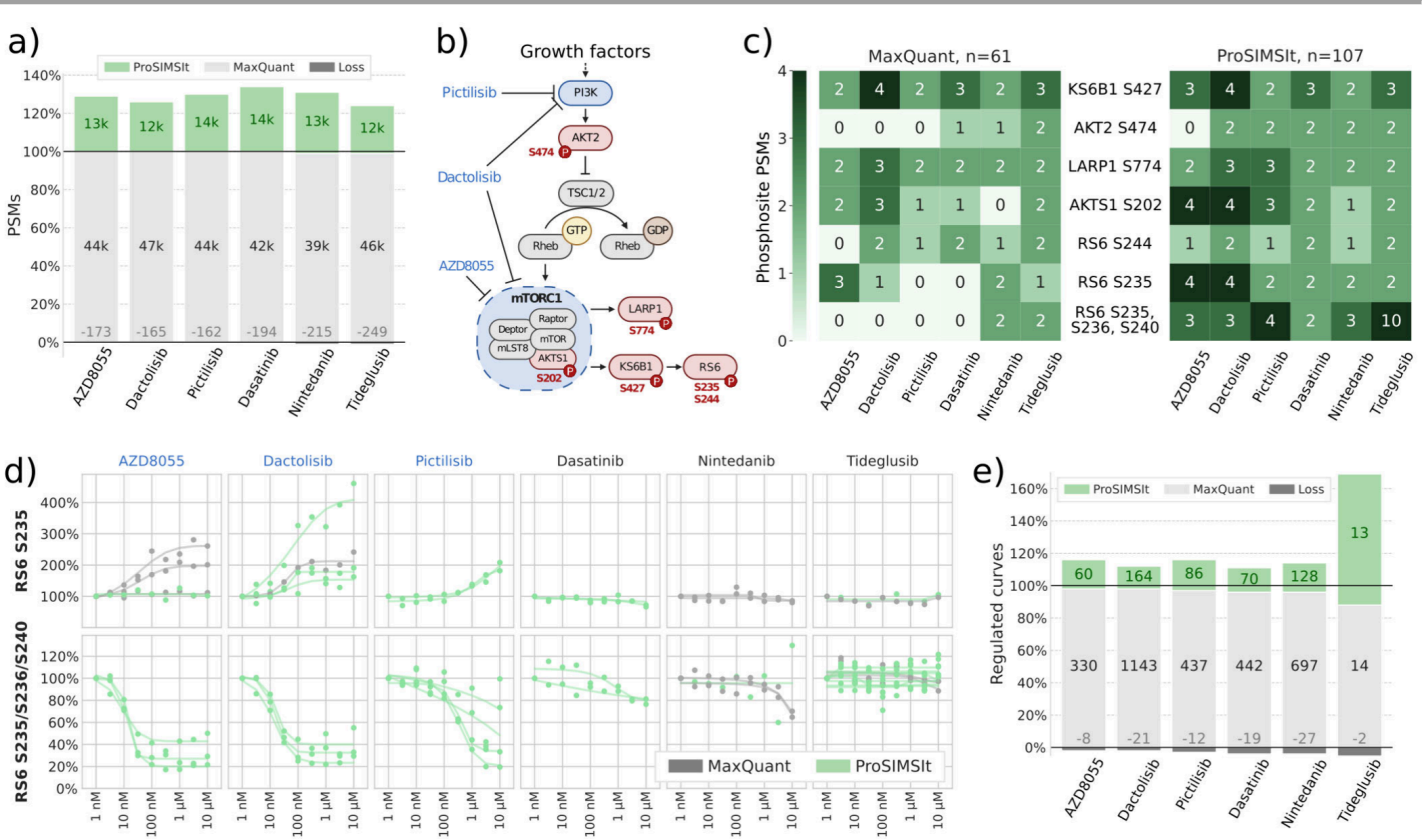
Applying ProSIMSIt on a decryptM data set. (a) Summary of PSMs identified by MaxQuant (gray) and ProSIMSIt (green) for each drug. (b) Simplified PI3K/AKT/mTOR pathway with highlighted phosphosites (red) and drugs with their respective targets (blue). (c) Heatmap of observed PSMs for highlighted phosphosites per drug from MaxQuant (left) and ProSIMSIt (right). (d) Curves of two peptides carrying the phosphosite RS6 S235, identified by MaxQuant (gray) and ProSIMSIt (green) for all drugs, with mTOR inhibitors highlighted in blue. (e) Overall gain in PSMs classified as up- or downregulated by CurveCurator.

In line with the earlier observed gains in the CPTAC phosphoproteome study, an average of 29% additional PSMs could be identified with ProSIMSIt for each drug (Figure 4a). To analyze the benefits of these gains for the biological interpretation, we specifically looked at phosphosites with known effector function in or associated with the PI3K/AKT/mTOR pathway, which should show the regulation of phosphosites for the three PI3K/mTOR inhibitors (Figure 4b). Across these sites of interest, ProSIMSIt increased the number of PSMs from 61 to 107. Notably, ProSIMSIt found PSMs for 10 out of the 11 drug–phosphosite combinations, for which MaxQuant did not find any PSM (Figure 4c). The phosphosite RS6 S235 was observed on various peptides, two of which were selected here to highlight how the additional PSMs from ProSIMSIt improve the interpretability of the data. This example highlights an exceptional strength of ProSIMSIt: inhibitory curves at medium to high potency are very valuable for interpreting drug mode of actions but are especially prone to eluding identification by the search engine. This is because the low abundance at higher doses leads to reduced combined precursor abundance resulting in lower-quality MS2 spectra. Here, this is illustrated by the fact that MaxQuant failed to identify the triply phosphorylated peptide for all three PI3K/mTOR inhibitors, but ProSIMSIt rescued 10 PSMs, all with down regulated curves. We can ascertain that these are true regulations by noting the upregulation of the single-phosphorylated version, a confounding effect caused by the inhibition of the phosphorylation on S236 (Figure 4d and Suppl. Figure S6). Complex interactions between phosphosites like the one unveiled here are highly valuable for decrypting drug mode of actions and benefit greatly from the additional curves rescued by ProSIMSIt. Additionally, ProSIMSIt may aid such investigations by facilitating the differentiation of positional isomers, by testing multiple candidate isomers for spectra with uncertain PTM localization through evaluation by Oktoberfest.

Across all drugs, ProSIMSIt substantially enhances the information content by identifying 14–68% additional regulated curves, each representing a potentially pivotal drug–phosphosite relation to decipher a drug’s mode of action (Figure 4e). Note that the 3% regulated curves lost by ProSIMSIt were generally not due to the loss of the PSMs. Rather, these curves were no longer classified as confidently regulated by CurveCurator due to differences in normalization.

## Conclusions

The ProSIMSIt pipeline substantially mitigates the missing value problem in multibatch isobaric labeling experiments, for example in clinical and pharmaceutical studies. The pipeline leverages the strengths of MS2 spectrum clustering via SIMSI-Transfer and data-driven rescoring via Oktoberfest. Here, we have shown its applicability to isobarically labeled global and phosphoproteome data sets. Nevertheless, the pipeline is directly compatible with other PTMs and fragmentation methods, such as multistage activation, but is dependent on the availability and efficacy of suitable peptide property prediction models.

On large-scale cancer cohort data as well as dose-response experiments, the added benefit is directly visible in the form of substantial increases in identified spectra as well as the discovery of previously unseen downstream effects of drug target inhibition. These examples underscore the potential of reanalyzing large-scale data sets with ProSIMSIt to uncover deeper biological insights, which is inviting for broader reanalysis of extensive isobaric-labeled data sets across the field.^29,30^ We anticipate that as the trend toward larger isobaric labeling multiplexing experiments continues, both in samples per batch^1^ and number of batches, ProSIMSIt will become an indispensable pipeline for large-scale studies of the future.

## Data Availability

The decryptM mass spectrometry proteomics data, the search engine results for all data sets, and the pipeline output have been deposited to the ProteomeXchange Consortium via the PRIDE^31^ partner repository with the data set identifier PXD057211. Previously published decryptM results can also be found via the data set identifier PXD037285. All CPTAC proteomics data for the endometrial carcinoma dataset are available via the CPTAC Proteomic Data Commons portal on https://proteomic.datacommons.cancer.gov/pdc/ through Proteomic Data Commons identifiers PDC000125 (global proteome) and PDC000126 (phosphoproteome).

## Supporting Information Available

Performance overview of the Prosit models utilized for phosphoproteome rescoring, detailed workflow of the ProSIMSIt pipeline, Percolator SVM score distributions of targets and decoys from ProSIMSIt results, results of entrapment experiment utilizing Percolator q-values, example mirror spectra with SIMSI-Transfer and Oktoberfest identifications, curves for all PSMs carrying the phosphosite RS6 S235, summary of intermediate and output files of ProSIMSIt, in-depth explanation of the ProSIMSIt algorithm, and entrapment experiment summary (PDF)
