# Supplementary material for: ProSIMSIt: The Best of Both Worlds in Data-Driven Rescoring and Identification Transfer
Source: J Proteome Res. 2025 Mar 22;24(4):2173–80. doi: 10.1021/acs.jproteome.4c00967 (PMC11976853; doi:10.1021/acs.jproteome.4c00967)
Supplement: Supplementary file 1 — pr4c00967_si_001.pdf [file pr4c00967_si_001.pdf]

Supporting Information for Manuscript: ProSIMSIt: The best of both worlds in data-driven rescoring and identification transfer

Firas Hamood[1], Wassim Gabriel[2], Pia Pfeiffer[2], Bernhard Kuster[1], Mathias Wilhelm[2], Matthew The*[1]

1 Chair of Proteomics and Bioanalytics, School of Life Sciences, Technical University of Munich, 85354 Freising, Germany
2 Assistant Professorship of Computational Mass Spectrometry, School of Life Sciences, Technical University of Munich, 85354 Freising, Germany

*Corresponding Author: Matthew The, +49 - (0)8161 - 714278, matthew.the@tum.de

Supplementary Figure S1: Performance overview of the Prosit models utilized for phosphoproteome rescoring.

Supplementary Text S1: Summary of intermediate and output files of ProSIMSIt

Supplementary Text S2: In-depth explanation of the ProSIMSIt algorithm

Supplementary Figure S2: Detailed workflow of the ProSIMSIt pipeline.

Supplementary Figure S3: Percolator SVM score distributions of targets and decoys from ProSIMSIt results.

Supplementary Text S3: Entrapment experiment summary

Supplementary Figure S4: Results of entrapment experiment utilizing Percolator q-values

Supplementary Figure S5: Example mirror spectra with SIMSI-Transfer and Oktoberfest identifications.

Supplementary Figure S6: Curves for all PSMs carrying the phosphosite RS6 S235.

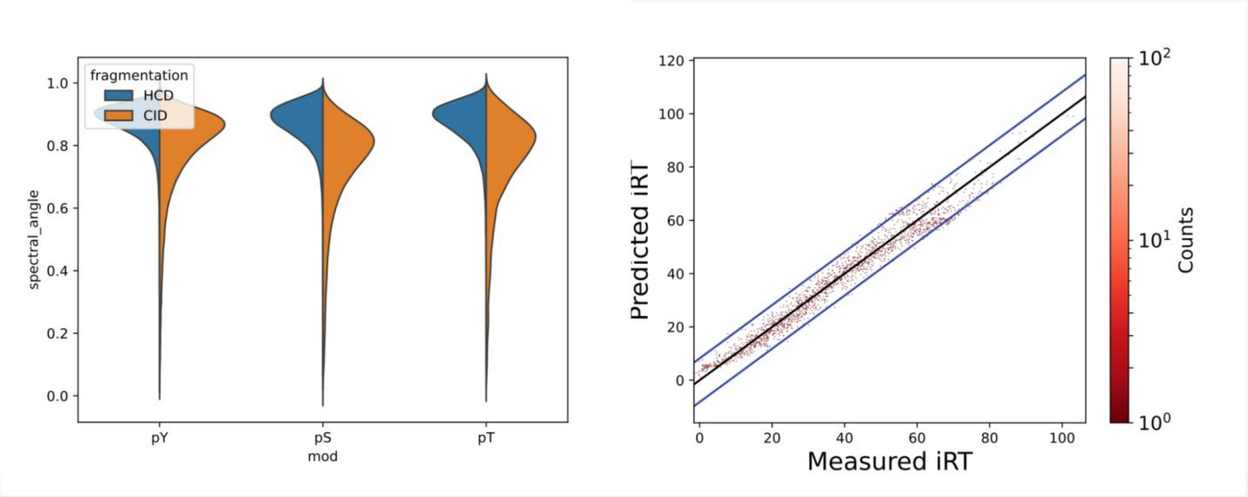

Supplementary Figure S1: Performance overview of the Prosit models utilized for phosphoproteome rescoring. Left: Spectral angle distribution between measured and predicted spectra of a ProteomeTools holdout dataset. Right: Scatter plot of measured and predicted retention time for each peptide of the same holdout dataset.

Supplementary Text S1: Summary of intermediate and output files of ProSIMSIt

- *MaxQuant*
    - msms.txt: Contains all identified PSMs with all identification characteristics, e.g. peptide sequence, score, and PEP
    - evidence.txt: Peptide-level output file, aggregating PSMs per raw file and charge state into peptides
    - allPeptides.txt: Contains all detected MS1 features and their characteristics, independent of successful identification
- *Prosit Peptide Property Prediction*
    - rescore.tab: PSM-level table of values for predicted and computed properties to be used as SVM features for Percolator
- *Percolator*
    - psms.txt: List of all target PSMs with their SVM score and q-values for FDR filtering
    - decoy.psms.txt: List of all decoy PSMs with their SVM score and q-values
    - weights.csv: Table of SVM features used by Percolator and their respective weights determined after the training step
- *SIMSI-Transfer*
    - p10_msms.txt: Similar structure as MaxQuant msms.txt, additionally containing PSMs generated by SIMSI-Transfer
- *Evidence assembly*
    - evidence_extended.txt: Similar structure as MaxQuant evidence.txt, additionally containing PSMs generated by SIMSI-Transfer
- *Picked Protein Group FDR*
    - updated_evidence.txt: Similar structure as MaxQuant evidence.txt with additional information generated by Picked Protein Group FDR
    - group_results.txt: Protein grouping output containing FDR filtered protein groups and additional protein-related information

Supplementary Text S2: In-depth explanation of the ProSIMSIt algorithm

1) Oktoberfest1
   Input files:
   - msms.txt: MaxQuant PSMs at 100% FDR
   - .raw spectrum files

   Processing steps:
   a) *Prosit Peptide Property Prediction*
      Prediction of MS2 spectra and comparison of measured with predicted spectra to generate various features to be used for Percolator, e.g. spectral angle.
   b) *Percolator*
      i) Training of SVM to optimally separate target PSMs from decoy PSMs.
      ii) PSM target-decoy competition to calculate FDR values.

   Output: psms.txt and decoy.psms.txt: PSM-level files for targets and decoys with q-values

2) SIMSI-Transfer
   Input files:
   - msms.txt: MaxQuant PSMs at 100% FDR
   - psms.txt: Percolator PSM output with q-values for FDR filtering
   - decoy.psms.txt: Percolator decoy PSM output with q-values for FDR filtering
   - .raw spectrum files

   Processing steps:
   a) Preprocessing *step* to filter msms.txt down to 1% FDR using Percolator results fromOktoberfest1; resulting file contains all PSMs that passed rescoring.
   b) *MaRaCluster*
      Cluster MS2 spectra based on similarity using a rarity-based distance metric.
   c) Merge step of cluster numbers with PSM information.
   d) *Identification transfer*
      Peptide identifications in clusters get transferred to unidentified spectra in that cluster. If multiple different peptides are identified within the same cluster, unidentified spectra generate one PSM for each peptide identified in the cluster.

   Output: p10_msms.txt: PSM-level output table structured similarly to MaxQuant msms.txt, with additional PSMs generated by SIMSI-Transfer; uncontrolled FDR, approximately 1%.

3) Oktoberfest2
   Input files:
   - p10_msms.txt: PSM-level output file from SIMSI-Transfer at approx. 1% FDR
   - rescore.tab: intermediate PSM file from previous Prosit Peptide Property Prediction at 100% FDR
   - weights.csv: SVM weights derived from Percolator training step in Oktoberfest1
   - .raw spectrum files

   Processing steps:
   a) Preprocessing step to filter for SIMSI-Transfer PSMs

To not perform peptide property prediction on many PSM twice, only PSMs of new identifications generated by SIMSI-Transfer are retained.

b) Prosit Peptide Property Prediction of transferred identifications only.
c) Merging step with remaining 100% FDR PSMs already processed with Prosit in Oktoberfest1, using previous rescore.tab file.
d) Percolator using weights.csv from Oktoberfest1
   i) No additional training is required, as the weights from the first Percolator run are re-used.
   ii) PSM target-decoy competition to calculate FDR values.

Output: psms.txt and decoy.psms.txt: PSM-level files with q-values containing final PSM-level results, can be filtered to 1% FDR. This file can be used for downstream processing if users are only interested in PSMs, e.g. for phosphoproteomic experiments.

4) Evidence assembly

Input files:

- psms.txt and decoy.psms.txt: Percolator PSM-level output files at 100% FDR from Oktoberfest2.
- evidence.txt: MaxQuant peptide-level identifications at 100% FDR.
- allPeptides.txt: MaxQuant MS1 feature list at 100% FDR.

Processing steps.

a) Building PSM-level file structured like MaxQuant msms.txt, containing posterior error probabilities used in the Picked Protein Group FDR step.
b) Assigning MS1 features to PSMs and aggregating to peptide level.

Output: evidence_extended.txt: Peptide-level file containing all peptide identifications generated during the workflow with 100% FDR.

5) Picked Protein Group FDR

Input files:

- evidence_extended.txt: Peptide-level file containing all identifications from ProSIMSIt
- .fasta database used for initial MaxQuant database search.

Output. updated_evidence.txt & group_results.txt: Peptide and protein group output files at 1% FDR.

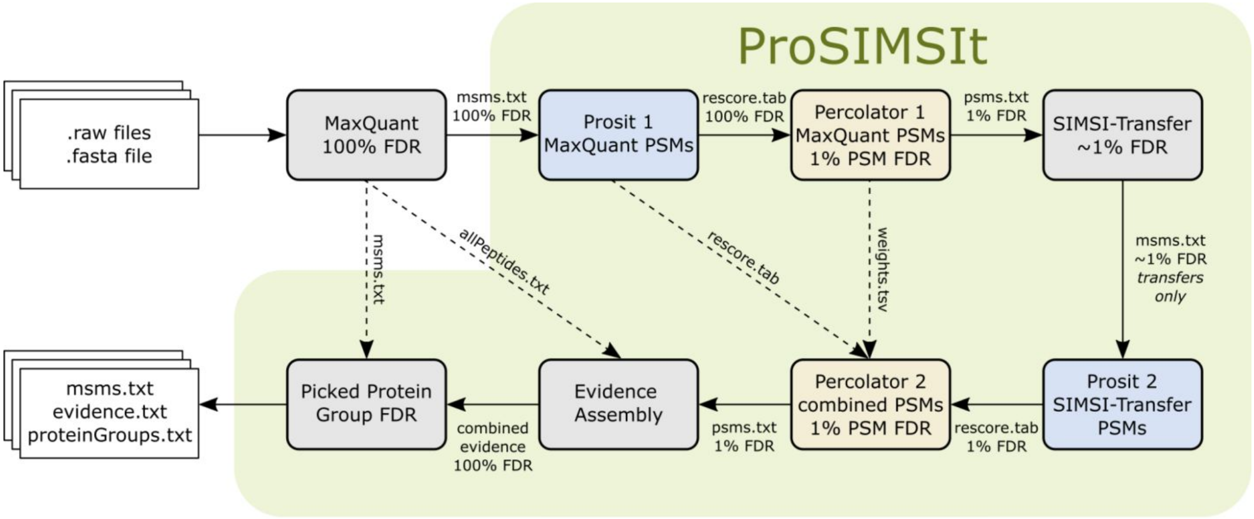

Supplementary Figure S2: Visualized summary of ProSIMSIt workflow. Files are first searched using MaxQuant without FDR filter. The resulting msms.txt file is provided to Oktoberfest, which first applies Prosit for fragment ion and retention time prediction. Additionally, various PSM features are generated and used as input for Percolator. Percolator trains an SVM to efficiently separate targets and decoys using the generated features. After training, the feature weights are stored in a weights.csv file and the dataset is re-scored and filtered for 1% FDR. The resulting file is used for SIMSI-Transfer, which utilizes MaRaCluster to cluster all MS2 spectra by similarity and transfers identifications across TMT batches. The newly generated PSMs are then used for a second Prosit run, generating the same features for Percolator. Prior to the second Percolator step, the two Prosit output files are merged. Additionally, the weights of the first Percolator run are used as a static model for the second run. This way, the newly generated PSMs by SIMSI-Transfer are evaluated in the same way as the initial MaxQuant PSMs. The resulting PSMs are used together with the MaxQuant allPeptides.txt file to generate a peptide-level evidence.txt file, which can be provided to Picked Protein Group FDR for protein assembly and FDR filtering to 1%.

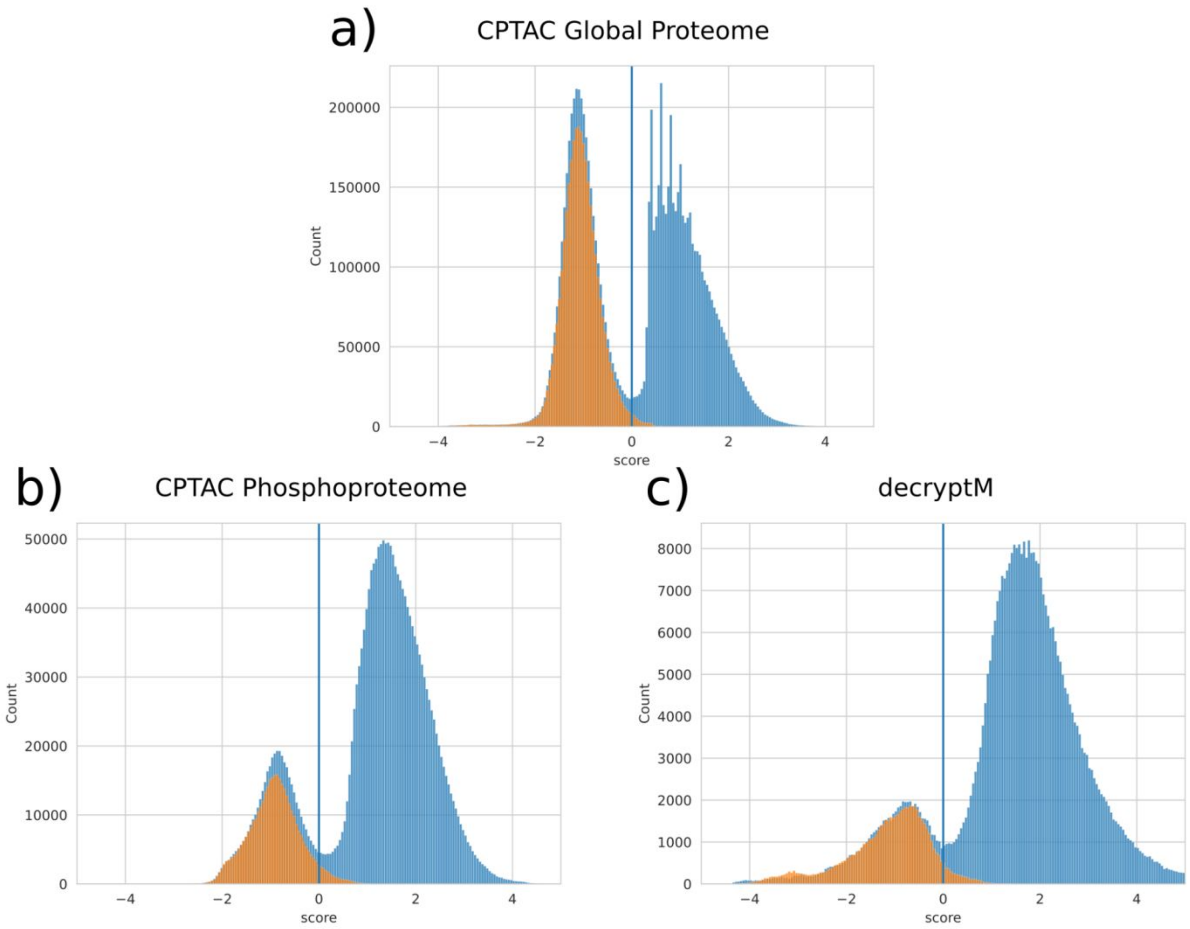

Supplementary Figure S3: Percolator SVM score distributions of targets (blue) and decoys (orange) from ProSIMSIt results for CPTAC global proteome (a), phosphoproteome (b), and decryptM (c) experiments. We observed a slight inflation of target hits compared to decoys at scores below 0, which is probably caused by a true positive peptide ID being transferred to an incorrect spectrum, causing a false positive.

Supplementary Text S3: Entrapment experiment summary

An entrapment database was generated from the previously used .fasta file using the open-source tool mimic (https://github.com/percolator/mimic/). A multiFactor of 4 was used (i.e. each protein sequence was used to generate four shuffled entrapment proteins), and the database was appended to the original .fasta database, generating an entrapment .fasta file 5x the original size. The exact same parameters as for the normal MaxQuant 100% run were used, except for allowing no missed cleavages as well as using the full .fasta headers as protein identifiers. The CPTAC global proteome dataset was searched against the entrapment database, and the resulting output files were used for ProSIMSIt processing. Entrapment analysis was performed for a) Percolator output files based on MaxQuant features, b) Percolator output files from the initial Oktoberfest run, and c) Percolator output files from the second Oktoberfest run in ProSIMSIt. This way, the eFDR for MaxQuant (a), Oktoberfest (b), and ProSIMSIt (c) was determined. eFDRs were calculated according to Wen et al.[1]:

lower limit $$eFDR_{low} = \frac{N_e}{(N_e+N_t)}$$

upper limit $$eFDR_{up} = \frac{N_e \times (1+\frac{1}{r})}{(N_e+N_t)}$$

where N_t and N_e denote the number of original target and entrapment discoveries, and r denotes the effective ratio of the entrapment database to the original target database (in our case, r=4).

The entrapment analysis showed that at 1% FDR, the eFDR lower limit was below 1% and the eFDR upper limit was above 1% for MaxQuant, Oktoberfest, and ProSIMSIt (Suppl. Fig. 4). ProSIMSIt showed a lower limit of 0.85% and an upper limit of 1.06% eFDR at 1% FDR, which is identical to Oktoberfest and near identical to MaxQuant (0.84% lower limit, 1.05% upper limit). This indicates a negligible increase compared to MaxQuant and Oktoberfest, confirming a proper FDR control of ProSIMSIt.

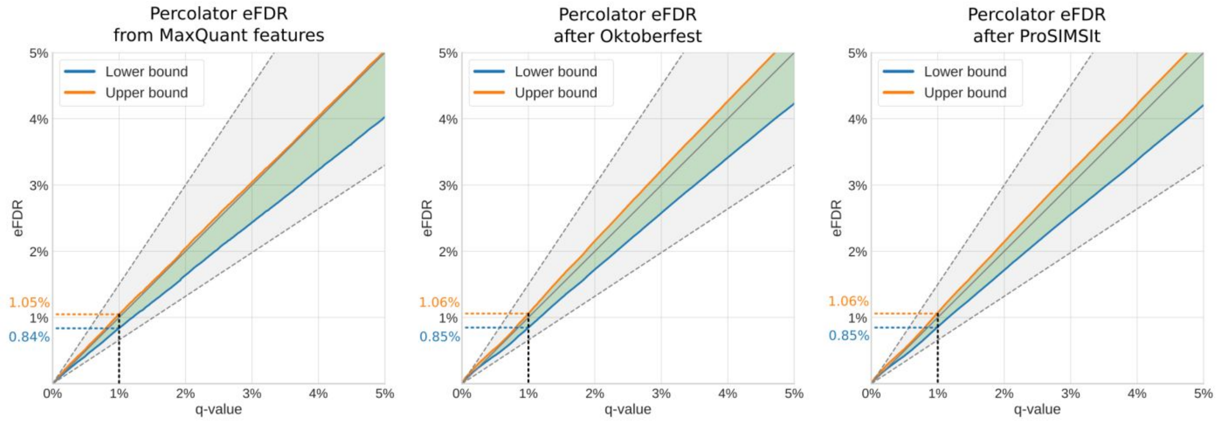

Supplementary Figure S4: Results of entrapment experiment utilizing Percolator q-values for MaxQuant (left), Oktoberfest (middle), and ProSIMSIt (right). All FDRs are calculated on PSM level.

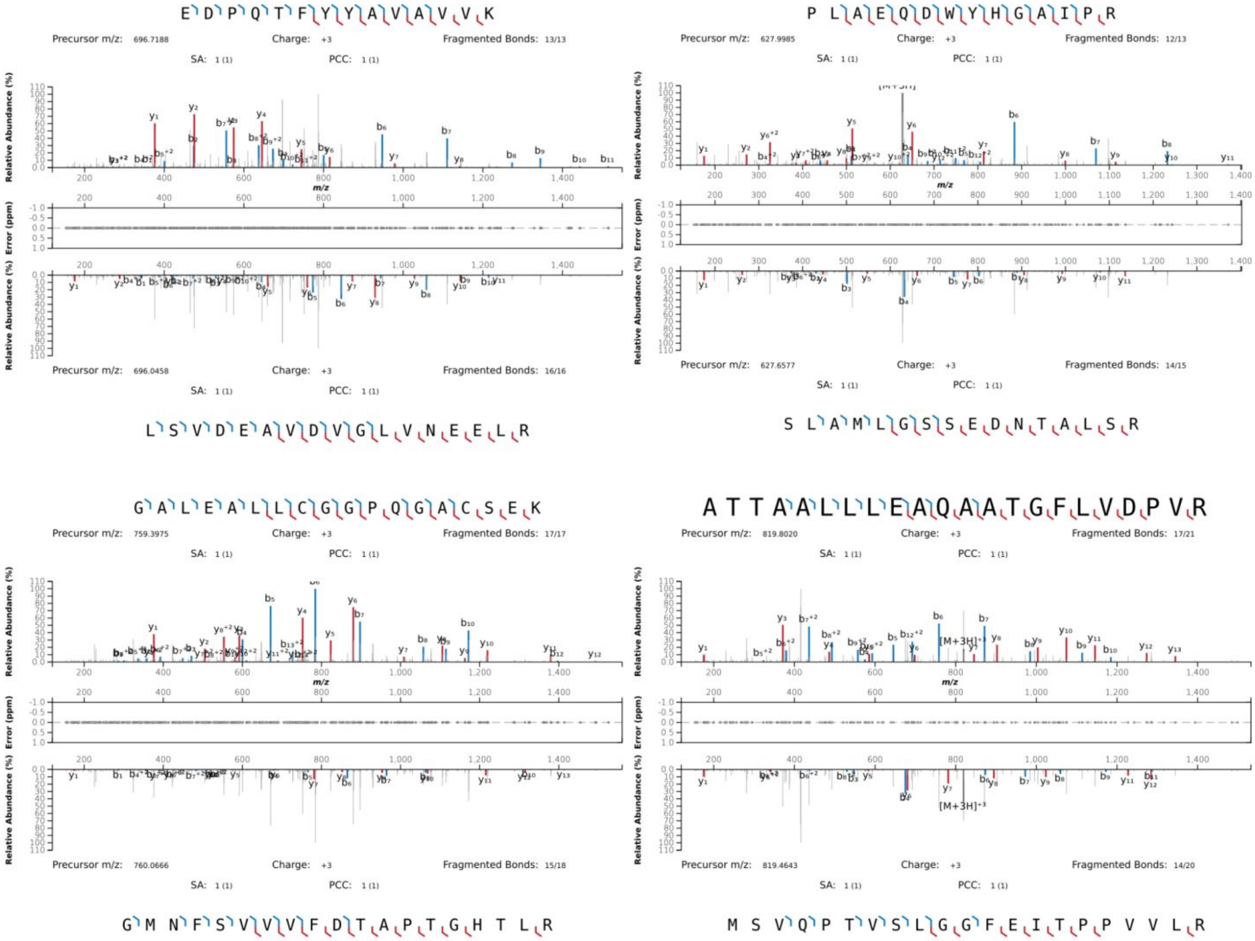

Supplementary Figure S5: Example mirror spectra with SIMSI-Transfer (top) and Oktoberfest (bottom) identifications. All shown spectra have a spectral angle of at least 0.8 between the measured spectrum and the predicted version of each peptide.

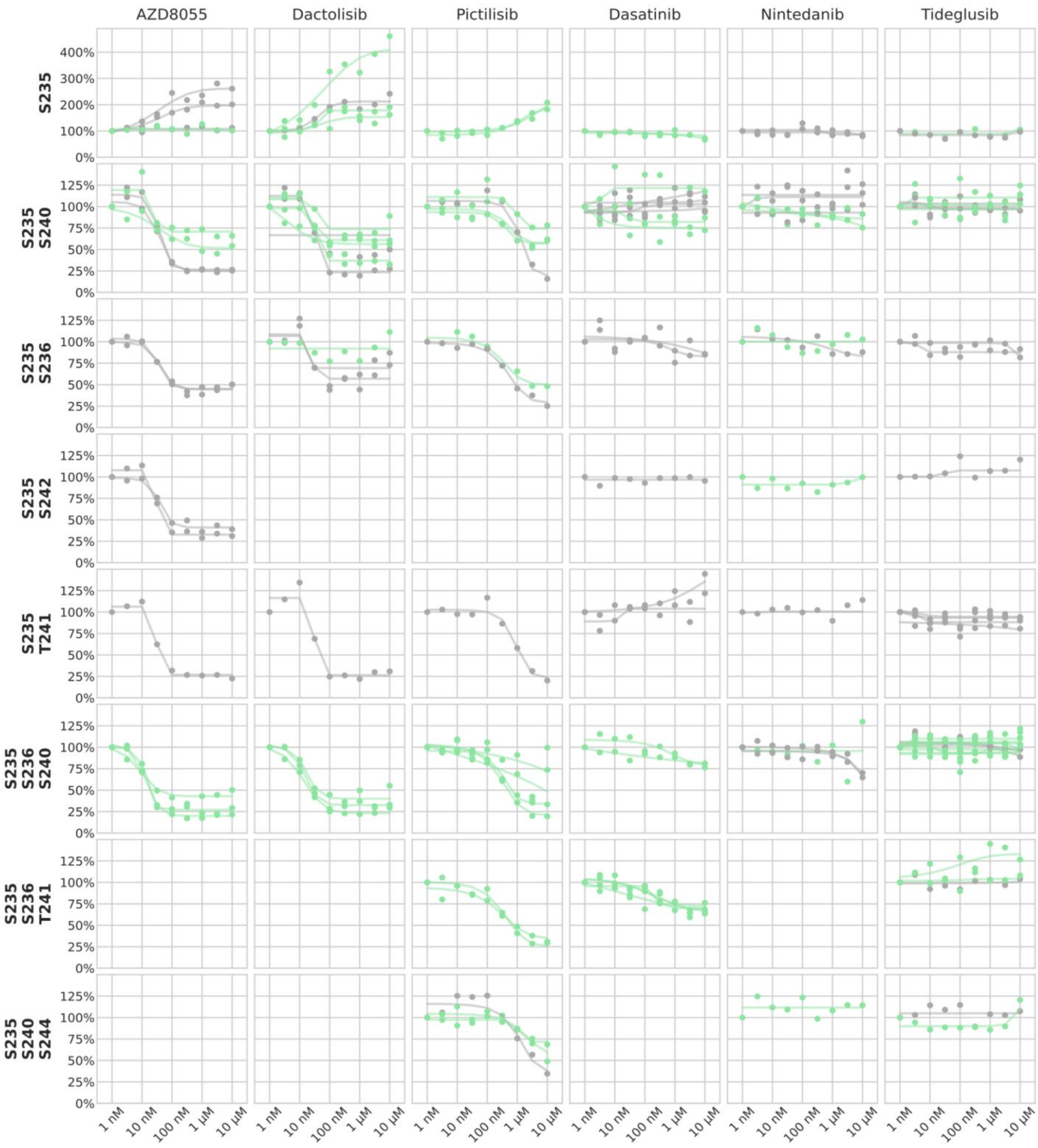

Supplementary figure S6: Curves for all PSMs carrying the phosphosite RS6 S235. Only peptides found in at least 3 of the 6 drug treatments are shown.

References

(1) Wen, B.; Freestone, J.; Riffle, M.; MacCoss, M. J.; Noble, W. S.; Keich, U. Assessment of false discovery rate control in tandem mass spectrometry analysis using entrapment. *bioRxiv : the preprint server for biology* [Online early access]. DOI: 10.1101/2024.06.01.596967. https://www.biorxiv.org/content/early/2025/01/21/2024.06.01.596967.
